# Moving Toward Objective Diagnosis in Fibromyalgia: Emerging Biomarkers and Digital Phenotyping Tools

**DOI:** 10.3390/biomedicines14020440

**Published:** 2026-02-15

**Authors:** Mario García-Domínguez

**Affiliations:** 1 Program of Immunology and Immunotherapy, CIMA-Universidad de Navarra, 31008 Pamplona, Spain; mgdom@unav.es; 2 Department of Immunology and Immunotherapy, Clínica Universidad de Navarra, 31008 Pamplona, Spain; 3 Centro de Investigación Biomédica en Red de Cáncer (CIBERONC), 28029 Madrid, Spain

**Keywords:** fibromyalgia, nociplastic pain, central sensitization, neuroinflammation, precision medicine, neuroimaging, multi-omics

## Abstract

Fibromyalgia is a complex chronic pain condition characterized by pervasive pain, persistent fatigue, and cognitive disturbances. Despite advances in understanding its neurobiological mechanisms, diagnosis largely relies on subjective symptom assessment and exclusion criteria, contributing to underdiagnosis and treatment delays. Recent research has increasingly focused on identifying objective biomarkers and leveraging digital phenotyping to improve diagnostic precision. Promising biomarkers include neuroimaging indicators of altered pain processing, neuroinflammatory signatures in cerebrospinal fluid and blood, and dysregulated neuroendocrine and autonomic patterns. In addition, metabolomics and transcriptomics have revealed molecular profiles associated with fibromyalgia pathophysiology. Concurrently, digital health tools (e.g., wearable sensors, ecological momentary assessment, and machine learning-based symptom clustering) offer opportunities for continuous, real-world data collection and individualized disease characterization. This body of work suggests that integrating biological and digital metrics could enable a transition from subjective to objective data-driven fibromyalgia classification, facilitating earlier diagnosis and improved therapeutic outcomes.

## 1. Introduction

**Figure 1:**
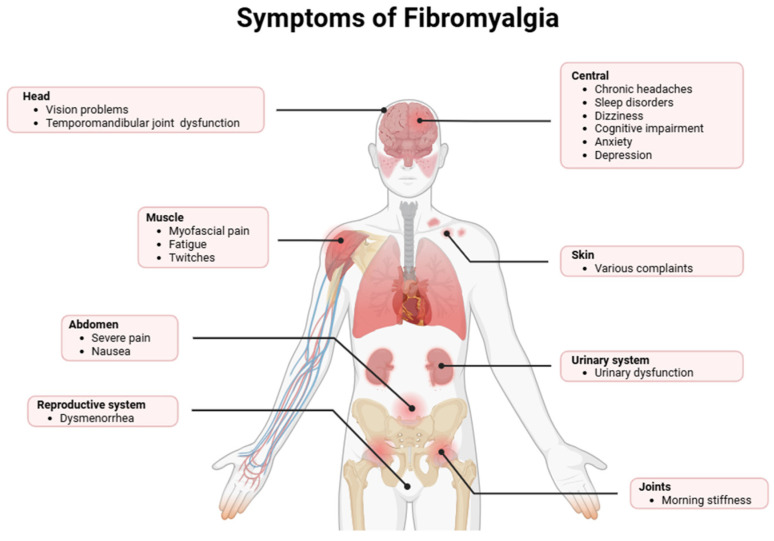
Fibromyalgia manifests with several clinical features, including CNS symptoms, cephalic and temporomandibular symptoms, widespread myofascial pain and fatigue, abdominal and genitourinary complaints, cutaneous dysesthesia, and peripheral joint stiffness. These manifestations reflect the diffuse and heterogeneous somatic and neuropsychiatric symptom burden that characterizes this chronic pain syndrome.

Fibromyalgia is a chronic disorder characterized by extensive nociplastic pain (arising from altered nociceptive processing without tissue damage or somatosensory lesions) accompanied by systemic, neurocognitive, and mood disturbances, resulting in significant functional and socioeconomic burden [1]. Epidemiological data shows a global prevalence ranging from 0.2% to 6.6%, with a marked female predominance, suggesting potential contributions of sex-specific neuroimmune and hormonal mechanisms [2,3]. The clinical phenotype is characterized by chronic widespread pain, severe fatigue, sleep architecture disruption, and executive and attentional deficits commonly described as “fibro-fog” [4]. Additional symptoms typically include pronounced nociceptive hypersensitivity (allodynia and hyperalgesia), musculoskeletal stiffness, cephalalgia, functional gastrointestinal disorders including irritable bowel syndrome (IBS), and high rates of psychiatric comorbidities, including anxiety and depressive disorders [5]. Emerging evidence also highlights dysregulation of the autonomic nervous system and altered central pain processing pathways, which may be linked to increased symptom severity and variability [6,7]. Moreover, chronic inflammation and immune system perturbations have been proposed as underlying mechanisms that exacerbate both peripheral and central manifestations of the disorder (Figure 1) [8,9].

This condition is progressively recognized as a central nociceptive processing disorder supported by maladaptive neuroimmune signaling within the central nervous system (CNS). Although its precise etiology remains elusive, converging evidence implicates maladaptive plasticity within pain-processing networks, characterized by impaired descending inhibitory pathways, enhanced excitatory neurotransmission, and sustained activation of glial cells [8]. These alterations may be precipitated or exacerbated by environmental stressors (e.g., physical trauma, infection, and chronic psychosocial stress) acting upon genetically or epigenetically susceptible individuals [9]. Neuroinflammation has emerged as a central pathophysiological component of fibromyalgia [10,11].

Beyond central mechanisms, accumulating evidence supports a significant contribution of peripheral neuroimmune alterations to fibromyalgia pathogenesis [12]. Peripheral nociceptors innervating skin and muscle tissues exhibit heightened excitability due to molecular sensitization of ion channels and receptors involved in pain transduction, such as transient receptor potential vanilloid (TRPV) channels, acid-sensing ion channels (ASICs), and purinergic P2X and P2Y receptors [13,14]. Some pro-inflammatory mediators released by immune cells within peripheral tissues further enhance nociceptor firing through cytokine-mediated signaling cascades, thus amplifying afferent nociceptive inputs [11,15].

The interplay between peripheral and central neuroimmune mechanisms forms a bidirectional feed-forward loop that drives both the initiation and maintenance of central sensitization [11]. Permanent peripheral nociceptive signaling can induce microglial priming and astrocyte reactivity in both the spinal cord and supraspinal structures, whereas CNS-derived inflammatory mediators can influence peripheral immune responses [15]. Central sensitization is associated with neurogenic inflammation, synaptic hyperexcitability, and maladaptive neuroplastic changes that underlie essential clinical features of fibromyalgia, including exaggerated tenderness, chronic pain amplification, peripheral edema, and cognitive dysfunction [16].

Given the multifactorial pathophysiology of fibromyalgia, it has become increasingly apparent that symptom-based clinical assessment alone is insufficient to delineate the heterogeneity of the disease phenotype. Accordingly, there has been an increasing focus on the development of supplementary diagnostic modalities designed to improve the clinical assessment [17]. These methodologies encompass neuroimmune biomarker analyses, advanced neuroimaging approaches, and neurophysiological evaluations, each providing a unique yet integrative insight into the fundamental pathophysiological processes [18]. At the molecular level, numerous studies have consistently reported elevated concentrations of several pro-inflammatory cytokines in cerebrospinal fluid (CSF) and skin tissues, such as IL-1β, IL-6, IL-8, IL-17, and TNF-α [19,20,21]. In addition to these pro-inflammatory mediators, chemokines including CCL2, CCL11, CXCL5, and CX3CL1 have been found to be increased, implicating enhanced leukocyte recruitment, glial activation, and neuroinflammatory signaling [22,23]. Anti-inflammatory cytokines, such as IL-10 and TGF-β, are often reported to be dysregulated, suggesting an impaired capacity for resolving neuroimmune activation [24,25]. Furthermore, peripheral mediators such as regulatory microRNAs (e.g., miR-4771 and miR-2115-3p) may contribute to peripheral neuroimmune interaction, thus reinforcing maladaptive pain processing [25]. Other factors, including substance P, are also increased in fibromyalgia patients, further potentiating peripheral and central sensitization through enhanced excitatory neurotransmission and glial activation [26].

On the other hand, advanced neuroimaging modalities have emerged as complementary tools for characterizing functional and structural aberrations within central pain-processing networks. Functional magnetic resonance imaging (fMRI) studies have repeatedly demonstrated hyperactivation of canonical nociceptive circuits (e.g., the insula, anterior cingulate cortex, and somatosensory cortices) upon nociceptive stimulation, concomitant with reduced engagement of descending inhibitory pathways, including those involving the periaqueductal gray and rostroventral medulla [27,28]. Diffusion tensor imaging (DTI) analyses have revealed microstructural abnormalities in white matter tracts implicated in pain modulation, such as the spinothalamic tract and corpus callosum, suggesting altered structural connectivity that may underpin abnormal pain processing [29]. Moreover, positron emission tomography (PET) using glial-specific tracers enables in vivo visualization of microglial and astrocytic activation, providing direct evidence of central neuroimmune dysregulation and glial contributions to chronic pain maintenance [30].

Finally, neurophysiological approaches serve as an additional layer of objective evaluation, permitting the quantification of nociceptive hyperexcitability and central sensitization. Quantitative sensory testing (QST) permits the systematic evaluation of thermal, mechanical, and vibratory detection and pain thresholds, thereby delineating the presence of allodynia, hyperalgesia, and other sensory disturbances that reflect the integrated contributions of peripheral and central mechanisms to symptom manifestation [31]. Pressure algometry provides additional objective indices of localized pain sensitivity, while evoked potential methodologies (e.g., laser-evoked and somatosensory evoked potentials) enable detailed temporal characterization of afferent nociceptive input and its cortical integration [32]. In parallel, microneurography offers high-resolution characterization of peripheral nerve fiber activity, associating aberrant afferent input with central hyperexcitability [33]. Collectively, these neurophysiological assessments link clinical symptomatology to quantifiable electrophysiological correlates, thereby strengthening the mechanistic understanding of fibromyalgia pathophysiology [27].

This review synthesizes current evidence on biological and digital biomarkers of fibromyalgia, highlighting their potential to enhance diagnostic accuracy. It discusses neuroimaging, neuroinflammatory, neuroendocrine, and autonomic markers, alongside molecular insights from metabolomics and transcriptomics, and examines digital phenotyping approaches. Through the convergence of these approaches, this review delivers a comprehensive synthesis of the field, delineates current knowledge gaps, and proposes future directions for objective diagnostics and personalized management of fibromyalgia.

## 2. Emerging Biological Biomarkers in Fibromyalgia

### 2.1. Neuroinflammatory and Immune Signatures

Growing evidence supports the concept that fibromyalgia is associated with measurable neuroinflammatory and immune-related alterations (Table 1), detectable both in CSF and peripheral blood [34,35]. Although fibromyalgia has long been classified as a non-inflammatory disorder, recent advances in immunological, proteomic, and transcriptomic research reveal a permanent low-grade neuroimmune activation profile [11]. These findings reinforce the hypothesis that immune signaling pathways contribute to central sensitization, pain amplification, fatigue, and cognitive dysfunction that characterize this disorder.

CSF analyses have shown dysregulated neuroimmune signaling within the CNS of individuals with fibromyalgia [34]. Among the most consistently reported findings is the increase in IL-8, which plays a key role in glial activation, neuronal excitability, and nociceptive transmission [36]. Increased CSF IL-8 levels have been interpreted as an indicator of persistent microglial and astrocytic activation, potentially sustaining central sensitization processes [37]. Beyond IL-8, elevated CSF concentrations of excitatory neurotransmitters, particularly glutamate, have been reported in patients with fibromyalgia, consistent with heightened excitatory signaling and impaired inhibitory pain modulation [38]. Proteomic profiling of CSF has further identified differential expression of numerous inflammation-related and metabolic proteins, including apolipoprotein C-III, galectin-3-binding protein, malate dehydrogenase, and proSAAS-derived peptides, implicating lipid metabolism, immune signaling, mitochondrial dysfunction, and neuropeptide processing in fibromyalgia pathophysiology [34,39]. Other emerging evidence shows the involvement of biomarkers indicative of subtle neuronal injury, including neurofilament light chain, may be altered in fibromyalgia patients, although this remains an area of investigation [40].

Peripheral blood studies have revealed a broad spectrum of immune abnormalities, reflecting systemic low-grade inflammation rather than classical autoimmune pathology [45]. Numerous studies have reported elevated circulating levels of pro-inflammatory cytokines, such as IL-6, IL-8, TNF-α, and IFN-γ [46]. These pro-inflammatory cytokines are recognized for modulating nociceptor sensitivity, synaptic plasticity, and neuroendocrine regulation, and their upregulation may underlie the widespread pain, fatigue, and sleep disturbances observed in fibromyalgia [47]. Acute-phase reactants, such as C-reactive protein (CRP) are also increased in several fibromyalgia patients, reinforcing the concept of chronic, low-intensity inflammation [48].

Beyond circulating cytokines, peripheral blood transcriptomic analyses have identified different immune gene expression signatures in fibromyalgia patients. Upregulation of interferon-regulated genes and inflammation-associated transcripts, including S100A8, S100A9, VCAM1, CD163, SERPINA1, and ANXA1, has been observed in B-cell populations [49]. These findings indicate chronic immune activation and dysregulated leukocyte function, which may impact neuroimmune communication via cytokine signaling and interactions with the blood–brain barrier (BBB) [50]. Proteomic platforms have additionally expanded the list of candidate serum biomarkers, identifying elevations in immune-modulatory proteins such as AXIN1, STAMBP, SIRT2, and additional chemokines and signaling molecules (CCL2, CCL17, CXCL9, and CXCL11), which are associated with symptom severity and disease burden [22,51].

Neurotrophic and neuroimmune signaling pathways appear to be altered in fibromyalgia. Brain-derived neurotrophic factor (BDNF), which plays a key role in synaptic plasticity and pain sensitization, is frequently elevated in serum and has been associated with intensified pain sensitivity and cognitive symptoms [41]. Alterations in substance P, nerve growth factor (NGF), along with other neuropeptides that communicate with the immune system, support a model in which peripheral immune activation and neuroinflammation converge to sustain nociplastic pain [42,43]. Importantly, correlations between peripheral inflammatory markers and CSF cytokine concentrations are generally weak, underscoring the huge complexity of immune signaling across the BBB and suggesting that peripheral biomarkers may partly reflect central neuroinflammatory processes [44].

### 2.2. Neuroendocrine and Autonomic Biomarkers

One of the most consistently reported findings in fibromyalgia is HPA axis dysregulation, primarily evidenced by abnormalities in cortisol secretion [52]. Numerous studies have documented flattened diurnal cortisol rhythms, with reduced morning cortisol levels, blunted cortisol awakening response (CAR), and reduced circadian amplitude [53,54,55,56]. These alterations suggest impaired adrenal responsiveness and disrupted central regulation at the level of the hypothalamus or pituitary [57]. Basal hypocortisolism, particularly under stress-free conditions, is usually observed and is thought to reflect chronic overexposure to stressors leading to an exhausted HPA axis phenotype [58]. Salivary, plasma, and urinary cortisol measurements often reveal lower total cortisol output across the day, alongside increased intraindividual variability [59,60,61].

At the molecular level, altered regulation of corticotropin-releasing hormone (CRH) has been shown in fibromyalgia patients [62]. Several patients show elevated basal CRH concentrations in CSF, despite normal or reduced peripheral cortisol levels, indicating impaired downstream signaling or adrenal insensitivity [63]. This dissociation suggests altered pituitary responsiveness to CRH, potentially mediated by changes in receptor sensitivity or post-receptor signaling pathways [64].

Dysregulation of glucocorticoid receptor (GR) signaling represents an additional key molecular biomarker domain. Reduced GR sensitivity has been proposed based on altered expression of GR-related genes and disrupted negative feedback control of the HPA axis [65]. Polymorphisms and epigenetic modifications in several genes, such as NR3C1, which encodes the GR, have been linked to fibromyalgia symptom severity and stress reactivity [66]. Aberrant DNA methylation patterns in GR-regulatory regions may contribute to persistent dysregulation of cortisol signaling, linking early-life stress and trauma to long-term neuroendocrine vulnerability in fibromyalgia patients [67].

In parallel to HPA axis alterations, fibromyalgia is associated with pronounced autonomic imbalance, typically characterized by sympathetic predominance and reduced parasympathetic activity [68]. Heart rate variability (HRV) studies consistently show reduced overall HRV, decreased high-frequency components, and elevated low-frequency to high-frequency ratios, reflecting reduced vagal tone and impaired autonomic adaptability [69]. This autonomic profile persists during stress exposure, indicating a chronic state of autonomic rigidity rather than situational dysregulation [70]. Catecholaminergic signaling is altered in fibromyalgia, with abnormalities in norepinephrine and epinephrine dynamics observed at both central and peripheral levels [71]. Reduced CSF norepinephrine levels, coupled with excessive sympathetic activation in response to many stressors, suggest impaired sympathetic regulation [72]. Altered sensitivity of adrenergic receptors, potentially β2-adrenergic receptors, mediate pain perception [73]. Genetic polymorphisms affecting genes encoding catechol-O-methyltransferase (COMT) are among the most robustly replicated genetic associations in fibromyalgia, associating impaired catecholamine metabolism with heightened pain sensitivity and stress vulnerability [74].

Autonomic dysregulation in fibromyalgia is further reflected in abnormalities of baroreflex sensitivity and cardiovascular control [75]. Dysregulated baroreceptor responsiveness contributes to orthostatic intolerance, dizziness, and exercise intolerance commonly reported by fibromyalgia patients [76]. Finally, dysregulation of melatonin has emerged as a crucial neuroendocrine biomarker in fibromyalgia patients [77]. Abnormal nocturnal melatonin secretion patterns have been shown in conjunction with non-restorative sleep, circadian rhythm disturbances, and increased pain sensitivity [78].

### 2.3. Omics-Based Biomarker Discovery

Omics technologies, particularly metabolomics and transcriptomics, have emerged as critical tools to unravel the molecular networks underpinning fibromyalgia, revealing a constellation of potential biomarkers and mechanistic insights (Table 2) [79,80]. Metabolomic profiling has shown alterations in some amino acid homeostasis, mostly branched-chain amino acids (BCAAs), leucine, isoleucine, and valine, which are frequently elevated, implicating disrupted skeletal muscle energy metabolism, impaired mitochondrial oxidative phosphorylation, and activation of mTOR signaling pathways [81]. Aromatic amino acids, such as phenylalanine, tyrosine, and tryptophan, display differential plasma and CSF levels, reflecting an imbalance in catecholaminergic and serotonergic neurotransmission [82,83]. Tryptophan metabolism is significantly dysregulated, with increased kynurenine, 3-hydroxykynurenine, quinolinic acid, and xanthurenic acid, alongside reduced serotonin and melatonin precursors, indicating enhanced neuroinflammatory signaling, excitotoxicity via NMDA receptor activation, and disruption of circadian regulation [84,85]. Glutamine and glutamate ratios are also disrupted, consistent with altered glutamatergic neurotransmission and central sensitization, while glycine and GABA levels suggest reduced inhibitory neurotransmission in central nociceptive circuits [86]. Arginine, citrulline, and ornithine imbalances point to dysregulated nitric oxide synthase (NOS) activity, endothelial dysfunction, and attenuated vasodilatory responses, which might contribute to tissue hypoperfusion and pain sensitization [87].

Lipidomic alterations are among the most consistently reported metabolic signatures in fibromyalgia. Elevated ceramides (C16:0, C18:0, C20:0), sphingomyelins (SM d18:1/16:0, SM d18:1/18:0), lysophosphatidylcholines (LPC 16:0, LPC 18:0), and phosphatidylcholines (PC 34:1, PC 36:2) suggest dysregulated sphingolipid and glycerophospholipid metabolism, which might modulate apoptosis, neuroinflammation, and membrane fluidity [88]. On the other hand, fibromyalgia patients showed significantly increased oxidative stress markers (TOS, TAS, MDA, and OSI) compared with controls, indicative of reduced oxidative lipid damage and deficient anti-inflammatory regulation [89]. Increased circulating acylcarnitines (C2-C18) denote incomplete fatty acid β-oxidation and mitochondrial dysfunction [90,91]. Moreover, disturbances in eicosanoid metabolism, like increased prostaglandin E2 (PGE2), thromboxane B2, and leukotriene B4, highlight activation of cyclooxygenase (COX) and lipoxygenase (LOX) pathways contributing to systemic and neuroinflammation [92]. Purine metabolism is also dysregulated, with increased plasma and cerebrospinal hypoxanthine, xanthine, uric acid, and inosine, suggesting augmented reactive oxygen species (ROS) production, oxidative stress, and nucleotide turnover [93,94]. TCA cycle intermediates, including citrate, α-ketoglutarate, succinate, fumarate, and malate, are dysregulated, pointing to mitochondrial dysfunction, compromised electron transport chain activity, and a metabolic reprogramming toward anaerobic glycolysis, as evidenced by increased lactate and pyruvate levels [95]. Increased creatine, creatinine, and guanidinoacetate levels suggest perturbed phosphagen metabolism, contributing to skeletal muscle fatigue and energy insufficiency [96].

Transcriptomic analyses reinforce these metabolic findings by identifying dysregulated genes in pathways governing immune activation, neurotransmission, mitochondrial function, oxidative stress response, and cellular metabolism. Peripheral blood mononuclear cell (PBMC) transcriptomes consistently reveal upregulation of proinflammatory cytokines and chemokines, including IL-6, IL-1β, TNF-α, IL-17, CCL2, CCL5, CXCL8, and CXCL10, accompanied by downregulation of anti-inflammatory mediators such as IL-10, TGF-β1, and SOCS3 [97]. Toll-like receptor (TLR) signaling genes, especially TLR2, TLR4, and MyD88, are also upregulated, suggesting an innate immune response [98,99]. Genes encoding mitochondrial respiratory chain components, such as ND4, CyB, NRF-1, Tfam, and UCP2, are consistent with impaired electron transport chain activity and reduced ATP production [100,101]. Glycolytic and fatty acid oxidation genes, including LDH, CPT1, and acyl-CoA dehydrogenases, are differentially expressed, supporting metabolomic evidence of altered energy metabolism [102]. Finally, several oxidative stress-response genes, such as SOD, GPX, NQO1, HO-1/HMOX1, are dysregulated in fibromyalgia patients, indicating upregulation and activation of oxidative stress-response pathways [103].

Conversely, altered expression of neurotransmission-related genes has been reported in fibromyalgia patients. Decreased expression of inhibitory neurotransmission genes, including GABRB3, is consistent with a central excitatory-inhibitory imbalance potentially underlying hyperalgesia and allodynia [104]. Finally, dysregulation of circadian rhythm- and stress-response-associated genes, like NR3C1, suggests a link between chronic stress, sleep disturbances, impaired proteostasis, and fibromyalgia pathophysiology [105].

The integration of metabolomic and transcriptomic signatures enables patient stratification and mechanistic insight while forming the basis for precision medicine [106]. This approach facilitates the identification of fibromyalgia endophenotypes and the development of targeted interventions [107]. Future studies integrating multi-omics data with longitudinal clinical phenotyping, neuroimaging, microbiome profiling, and machine-learning approaches will be crucial to refine molecular networks, validate robust biomarkers, and translate molecular findings into diagnostic, prognostic, and therapeutic tools for fibromyalgia [106,108,109,110].

## 3. Digital Phenotyping Approaches in Fibromyalgia

### 3.1. Neuroimaging Markers of Altered Pain Processing

Fibromyalgia is increasingly recognized as a disorder of central pain processing rather than merely peripheral nociception, and neuroimaging studies have been pivotal in elucidating the neural substrates underlying its clinical manifestations [111]. Functional magnetic resonance imaging (fMRI) studies consistently demonstrate abnormal activation patterns in response to both experimentally induced and spontaneous pain [112]. Hyperactivation of the insular cortex, particularly the anterior insula, reflects heightened interoceptive and affective processing of nociceptive signals, whereas increased activity in the anterior cingulate cortex (ACC) is associated with enhanced emotional appraisal and salience attribution to painful stimuli [113,114,115,116]. Simultaneously, hypoactivation or reduced modulation within the prefrontal cortex (PFC) and periaqueductal gray (PAG) suggests impaired engagement of top-down inhibitory circuits, which normally attenuate pain perception [117,118].

Resting-state fMRI analyses provide complementary insights by revealing intrinsic network abnormalities independent of stimulus presentation [119]. Increased functional connectivity between the default mode network (DMN) and key pain-processing regions, including the insula and ACC, has been linked to persistent rumination on pain and maladaptive attentional biases [120]. Simultaneously, reduced connectivity between the DMN and executive control networks, especially within dorsolateral prefrontal and parietal cortices, correlates with cognitive impairments frequently reported in fibromyalgia patients, including deficits in working memory, cognitive flexibility, and attentional control [121]. Altered salience network connectivity, encompassing the ACC and anterior insula, further reflects abnormal detection and prioritization of sensory inputs, suggesting that fibromyalgia patients exhibit increased sensitivity not only to nociceptive stimuli but also to environmental stressors that may exacerbate pain perception [122].

Structural neuroimaging has revealed convergent evidence of gray and white matter abnormalities. Voxel-based morphometry (VBM) studies report reductions in gray matter volume in regions integral to pain and affective processing, such as the ACC, insula, thalamus, and prefrontal cortices [123,124]. These changes are interpreted as neuroplastic adaptations to chronic nociceptive input, potentially reflecting maladaptive sensitization or excitotoxicity [125]. Diffusion tensor imaging (DTI) further identifies microstructural alterations in white matter pathways connecting the PFC, limbic system, and somatosensory regions, implicating disruptions in circuits mediating top-down modulation of pain and affective responses [126].

Progress in multimodal neuroimaging has enhanced our understanding of underlying mechanisms. Magnetic resonance spectroscopy (MRS) studies indicate region-specific neurotransmitter imbalances, such as elevated glutamate levels in the insula and decreased GABA contents in prefrontal regions [127]. Positron emission tomography (PET) imaging has also demonstrated altered μ-opioid receptor availability in the ACC, PFC, and amygdala, further supporting the notion of impaired endogenous analgesic mechanisms [128].

### 3.2. Wearable Technologies and Continuous Physiological Monitoring

The advent of wearable technologies has revolutionized the study and management of fibromyalgia. Contemporary wearable devices, including wrist-worn actigraphy units, chest straps, smart textiles, and adhesive biosensors, allow for the continuous, high-resolution collection of physiological data encompassing physical activity, sleep architecture, and autonomic nervous system function [129]. Integrated accelerometry and gyroscopic sensors allow detailed measurement of daily movement patterns, capturing step counts, activity levels, postural changes, and periods of inactivity [130]. In fibromyalgia, where patients often modulate activity to avoid pain exacerbation, these continuous metrics permit objective assessment of low-level activity fluctuations and fatigue accumulation that persist unseen by retrospective self-reports or clinical observation [131,132]. Beyond simple activity measures, these devices enable the correlation of physical behavior with pain flares, offering insight into the bidirectional interactions between activity limitation, energy expenditure, and symptom severity [133].

Sleep disturbances, a pivotal component of fibromyalgia pathology, can be evaluated with unparalleled granularity through wearable technologies combining actigraphy with photoplethysmography, skin temperature, and electrodermal activity sensors [134,135]. Continuous monitoring captures sleep onset latency, nocturnal awakenings, sleep fragmentation, and sleep-stage distribution, including non-rapid eye movement (NREM) and rapid eye movement (REM) phases [136]. This is relevant in fibromyalgia, where disruptions in slow-wave sleep are implicated in central sensitization and impaired descending pain inhibition [137]. Integration of sleep and activity data allows researchers to model circadian patterns and examine the temporal relationships among daytime fatigue, nocturnal arousals, and autonomic fluctuations. Such analyses provide mechanistic insight into the interplay between restorative sleep deficits and symptom exacerbation, potentially informing targeted interventions [138,139].

Autonomic nervous system monitoring through wearable sensors, including continuous HRV, skin conductance, peripheral temperature, and respiratory rate, reveals physiological dysregulation frequently observed in fibromyalgia [140]. Altered sympathetic-parasympathetic balance, manifesting as reduced HRV, heightened sympathetic tone, and blunted parasympathetic responses, correlates with heightened pain perception, reduced stress resilience, and impaired sleep quality [68]. Prolonged, multimodal monitoring permits temporal mapping of autonomic fluctuations relative to activity and sleep patterns, revealing periods of vulnerability that might precede pain flare-ups or cognitive fatigue [141]. Furthermore, the application of advanced signal processing and machine learning to these datasets allows predictive modeling of symptom trajectories, providing a framework for personalized and proactive management strategies [142].

Despite the potential of wearable technologies, some limitations merit careful consideration. Sensor accuracy can be influenced by device placement, motion artifacts, and inter-individual variability in skin characteristics, cardiovascular responsiveness, or body composition, potentially confounding heart rate, electrodermal, and temperature measurements [143,144]. Patient adherence is an essential concern, as chronic pain, fatigue, tactile hypersensitivity, or discomfort from prolonged device wear may reduce compliance, introducing biases in data acquisition [145]. The interpretation of continuous, multimodal data in fibromyalgia is further complicated by the heterogeneous nature of the disorder, necessitating longitudinal baselines and analytical frameworks to distinguish pathological alterations from normal physiological variability [106,142]. Moreover, clinical integration of wearable-derived data prompts practical and ethical considerations, such as data privacy, storage, interoperability with electronic health records, and the need for actionable insights to facilitate therapeutic strategy [146]. Addressing these challenges is essential to ensure that wearable technologies provide not only precise and ecologically reliable measurements and actionable insights for patient-specific management planning, mechanistic understanding, and ultimately the enhancement of quality of life for fibromyalgia patients.

### 3.3. Ecological Momentary Assessment and Real-World Symptom Capture

Ecological Momentary Assessment (EMA) is a methodological approach particularly well suited to the study of fibromyalgia [147]. This method involves the real-time collection of data in participants’ natural environments, typically through brief questionnaires, mobile applications, or electronic devices. EMA captures experiences, symptoms, and behaviors as they occur in daily life, thereby reducing the recall bias inherent in retrospective self-reports [148]. Traditional retrospective assessments, often administered during clinical visits, are impaired by recall bias, temporal averaging, and an underrepresentation of real-world contexts [149]. Traditional retrospective assessments, often conducted through questionnaires during clinic visits, are limited by recall bias, temporal averaging, and abstraction from real-world contexts [150].

EMA has been particularly informative in elucidating the complex temporal dynamics of fibromyalgia symptoms. Pain, fatigue, cognitive dysfunction (“fibro fog”), sleep disturbances, and affective symptoms fluctuate not only across days but also within the same day. High-frequency EMA sampling has demonstrated meaningful diurnal patterns, such as morning stiffness, transient mid-day symptom relief, and evening symptom worsening, which are often masked by daily or weekly summary measures [150]. Capturing these rhythms provides insight into potential links with circadian regulation, sleep quality, autonomic functioning, and daily activity cycles, and helps explain why patients with similar average symptom levels might experience very different degrees of functional impairment [138].

Beyond diurnal variation, EMA enables the examination of short-term symptom instability, an increasingly recognized feature of fibromyalgia [151]. Moment-to-moment symptom variability and abrupt exacerbations have been associated with central sensitization, emotional dysregulation, and maladaptive coping strategies. Importantly, EMA-derived indices of symptom volatility might capture disease burden more accurately than mean symptom intensity and appear to be closely associated with disability, perceived lack of control, and reduced quality of life [152].

EMA is also uniquely suited to examining the contextual sensitivity of fibromyalgia symptoms. Fibromyalgia symptoms are dynamically modulated by many environmental, behavioral, and psychosocial factors, like physical activity, stress exposure, social interactions, affective states, and sleep quality. By measuring symptoms in close temporal alignment with contextual variables, EMA supports the identification of real-world symptom triggers and protective factors [153].

The relationship between physical activity and fibromyalgia symptoms illustrates the added value of EMA. Although exercise is widely recommended, patients usually report activity-related symptom exacerbation [154]. EMA studies, integrated with wearable sensors, demonstrate that activity-symptom relationships are highly context-dependent and influenced by factors like baseline fatigue, emotional state, prior sleep, and pacing strategies [155]. These individualized activity-symptom contingencies are difficult to detect using traditional methods but are essential for developing personalized rehabilitation and self-management interventions [156].

Cognitive and affective symptoms, including concentration difficulties, anxiety, and depressive affect, also show substantial variability [157]. EMA research suggests that these symptoms often co-vary with pain and fatigue but may also fluctuate independently, especially in response to stress or cognitive load. EMA designs enable the modeling of short-term, bidirectional relationships between symptoms and affect, contributing to a more refined understanding of symptom maintenance mechanisms [158].

### 3.4. Machine Learning and Data-Driven Patient Stratification

Machine learning (ML) enables systematic analysis of the complex heterogeneity inherent in fibromyalgia [94]. Conventional analytical approaches usually fail to capture the nonlinear interactions among symptoms, whereas ML combines high-dimensional clinical, psychosocial, behavioral, and biological data to delineate patient subgroups and characterize symptom trajectories [159].

Unsupervised learning, mainly symptom clustering through methods like k-means, hierarchical clustering, and Gaussian mixture models, consistently demonstrates that fibromyalgia encompasses heterogeneous phenotypes differing in pain intensity, fatigue, sleep disturbances, mood, and functional impairment, often independent of demographic variables [160]. Predictive modeling approaches, such as random forests, gradient boosting, and neural networks, permit prediction of outcomes including symptom severity, loss of function, quality of life, and treatment response [161]. Incorporating longitudinal data via recurrent neural networks or temporal convolutional models captures intra-individual symptom fluctuations, providing early warning signals and informing personalized interventions [162]. Integrating unsupervised clustering with supervised predictive modeling enhances clinical relevance by refining phenotypic stratification and prognostic prediction [163].

## 4. Clinical Implications and Translational Potential

Patient stratification constitutes a pivotal component in optimizing therapeutic outcomes in fibromyalgia [164]. This disorder manifests pronounced phenotypic heterogeneity, which can differentially influence disease progression and therapeutic response [165]. Consequently, conventional one-size-fits-all therapeutic approaches are intrinsically limited, underscoring the need for precision medicine frameworks that integrate systematic, multidimensional patient stratification [166].

Pharmacologic interventions in fibromyalgia exhibit substantial interindividual variability, consistent with underlying mechanistic heterogeneity. Serotonin-norepinephrine reuptake inhibitors (SNRIs), including duloxetine and milnacipran, modulate descending inhibitory pain pathways and often show enhanced efficacy in patients exhibiting prominent affective dysregulation and central sensitization [167]. Gabapentinoids, like pregabalin and gabapentin, regulate VGCCs to attenuate excitatory neurotransmission, showing benefit in individuals with hyperalgesia or allodynia [168]. Tricyclic antidepressants provide analgesic and sleep-modulatory effects, though their anticholinergic profile can limit tolerability, particularly in older adults [169]. Novel pharmacologic approaches, including low-dose naltrexone, act by attenuating microglial activation and neuroinflammatory cascades, offering potential therapeutic avenues for patients with heightened central immune signaling [170]. These observations illustrate the importance of mechanistically informed treatment selection to improve clinical outcomes and reduce the reliance on empiric trial-and-error approaches.

Non-pharmacologic interventions also demonstrate variable effectiveness according to individual neuropsychological and psychosocial profiles. Cognitive-behavioral therapy (CBT) targets maladaptive mental appraisals and coping strategies [171], while mindfulness-based stress reduction (MBSR) interventions potentiate parasympathetic tone, emotional resilience, and attentional control [172]. Graded exercise programs (GEP) involve dose titration tailored to baseline fatigue, pain sensitivity, and cardiovascular function to maximize benefit while minimizing symptom exacerbation [173,174]. Neurofeedback and other neuromodulation techniques aim to normalize aberrant cortical network activity associated with central pain processing and emotional regulation [175]. Integration of these behavioral and neuromodulatory strategies with pharmacologic interventions allows a multidimensional, personalized approach, where treatment is matched to both phenotypic and mechanistic characteristics, ultimately improving functional outcomes [166].

Translational research in fibromyalgia increasingly focuses on targeting mechanistic pathways implicated in symptom generation and maintenance. Central sensitization, reflected in hyperexcitability of nociceptive neurons and impaired inhibitory control within the CNS, remains a primary focus for therapeutic modulation [176]. Neuroinflammatory processes, mediated by glial activation, pro-inflammatory cytokine release, and dysregulated neuroimmune signaling, constitute an additional target for therapeutic intervention [45]. Mitochondrial dysfunction and metabolic dysregulation contribute to fatigue, muscular pain, and cognitive impairment, while peripheral sensitization, including small fiber neuropathy, might drive widespread hyperalgesia in specific patient subsets [177]. Investigational pharmacologic agents, including glial modulators, ion channel regulators, neuroimmune signaling inhibitors, and metabolic enhancers, aim to correct these pathophysiological derangements [178]. Adjunctive neuromodulation strategies, including transcranial magnetic stimulation, transcutaneous electrical nerve stimulation, and vagus nerve stimulation, support mechanistically informed approaches to restore central pain circuitry and autonomic function [179,180].

Conversely, the incorporation of molecular, neuroimaging, and electrophysiological biomarkers into clinical trials holds considerable promise for improving the rigor and precision of therapeutic assessment. Biomarker-guided enrichment strategies can identify homogeneous patient cohorts, thereby improving signal detection in clinical trials and supporting mechanistic validation of novel interventions [181]. Biomarker-driven enrichment strategies can define mechanistically homogeneous patient cohorts, improving signal detection in trials and facilitating mechanistic validation of novel interventions [182]. When combined with patient-reported outcomes, objective physiological data facilitate a multidimensional framework for assessing treatment response, guiding individualized therapy adjustments, and refining predictive stratification models [183]. Such strategies adhere to the principles of precision medicine, focusing on targeted interventions customized to the biological and phenotypic characteristics of each patient.

Despite these advancements, significant barriers to clinical translation persist. Methodological heterogeneity, small sample sizes, and insufficient longitudinal validation restrict the applicability of candidate biomarkers and patient stratification algorithms [17].

High costs and technical complexity associated with molecular diagnostics and advanced neuroimaging restrict widespread accessibility, resulting in disparities in care [108]. Psychosocial factors, including variability in symptom expression, stigma, and clinician skepticism, further worsen diagnosis, patient engagement, and treatment adherence [184,185]. Ethical considerations related to predictive biomarker use, risk stratification, and equitable access to next-generation diagnostics and therapeutics necessitate careful attention to patient autonomy, consent, and equity in healthcare delivery [186]. Addressing these challenges requires multicenter, longitudinal studies, standardized measurement protocols, cost-effective diagnostic platforms, clinician education, and proactive patient engagement initiatives.

## 5. Conclusions

Fibromyalgia represents a paradigmatic nociplastic pain disorder, characterized by widespread musculoskeletal pain, debilitating fatigue, cognitive deficits, and autonomic imbalance, arising from interactions between central and peripheral neuroimmune, neuroendocrine, and metabolic mechanisms. Converging evidence from molecular, neuroimaging, and neurophysiological studies shows chronic low-grade neuroinflammation, maladaptive central sensitization, and dysregulated neurotransmission as pathophysiological substrates. Peripheral immune response, aberrant nociceptor excitability, and metabolic perturbations further contribute to symptom amplification and maintenance, establishing a bidirectional feed-forward loop between the periphery and CNS.

Advances in omics technologies, including metabolomics and transcriptomics, have elucidated disrupted energy metabolism, oxidative stress pathways, and neurotransmitter imbalances, enabling the identification of candidate molecular biomarkers for disease stratification and mechanistic insight. Neuroimaging modalities, such as fMRI, PET, DTI, and MRS, have consistently demonstrated functional and structural abnormalities in pain-processing networks, supporting the central sensitization model and revealing potential targets for therapeutic modulation. Additionally, wearable devices, ecological momentary assessment, and machine learning approaches have facilitated high-resolution, real-world characterization of symptom dynamics, highlighting the temporal variability, contextual sensitivity, and phenotypic heterogeneity inherent to fibromyalgia.

These findings underscore the shortcomings of symptom-based diagnostics and emphasize the need for integrative, multidimensional frameworks that incorporate molecular, neuroimaging, neurophysiological, and digital physiological monitoring data. Patient stratification based on mechanistic and phenotypic signatures holds significant potential for precision medicine, supporting targeted pharmacologic and non-pharmacologic interventions, optimized monitoring, and improved functional outcomes. Nevertheless, challenges including methodological heterogeneity, limited longitudinal validation, accessibility constraints, and ethical considerations must be addressed to translate these discoveries into routine clinical practice. In addition, substantial real-world barriers hinder implementation within healthcare systems, such as high economic costs, limited infrastructure and technological resources, restricted access in low-resource settings, and variable acceptability among patients and healthcare professionals due to concerns about usability, interpretability, training requirements, and clinical workload integration.

Collectively, the integration of multi-omics, neuroimaging, and digital phenotyping approaches constitutes a transformative paradigm for advancing fibromyalgia research and clinical management. Future efforts should focus on longitudinal, multimodal studies, mechanistically informed patient stratification, and personalized intervention strategies to reduce diagnostic delays, enhance treatment efficacy, and improve quality of life for individuals affected by this debilitating disorder. Equally important will be the development of scalable, cost-effective, and ethically robust implementation models that promote equitable access and facilitate clinical adoption, confirming that these innovations translate into sustainable, real-world healthcare solutions.

## Figures and Tables

**Table 1:** Central and peripheral biomarkers associated with neuroinflammation, immune dysregulation, and nociplastic pain mechanisms. Abbreviations: CSF (cerebrospinal fluid); IL-8 (interleukin-8); BDNF (brain-derived neurotrophic factor); NGF (nerve growth factor); IL-6 (interleukin-6); TNF-α (tumor necrosis factor alpha); IFN-γ (interferon gamma); CRP (C-reactive protein); S100A8 (S100 calcium-binding protein A8); S100A9 (S100 calcium-binding protein A9); VCAM1 (vascular cell adhesion molecule 1); CD163 (cluster of differentiation 163); SERPINA1 (serpin family A member 1); ANXA1 (annexin A1); AXIN1 (axis inhibition protein 1); STAMBP (STAM binding protein); SIRT2 (sirtuin 2); CCL2 (C-C motif chemokine ligand 2); CCL17 (C-C motif chemokine ligand 17); CXCL9 (C-X-C motif chemokine ligand 9); CXCL11 (C-X-C motif chemokine ligand 11); and BBB (blood–brain barrier).

Source	Biomarkers	Functions	References
CSF	Elevated IL-8 and glutamate levels Altered expression of neurofilament light chain, apolipoprotein C-III, galectin-3-binding protein, malate dehydrogenase, and pro-SAAS-derived peptides	Microglial and astrocytic activation Increased neuronal excitability Enhanced nociceptive transmission Altered lipid metabolism Mitochondrial and neuropeptide processing dysfunction	[36,37,38,39,40]
	Increased BDNF, substance P, and NGF levels	Enhanced synaptic plasticity Increased pain sensitization Convergence of peripheral immune activation and central neuroinflammation sustaining nociplastic pain	[41,42,43,44]
Peripheral blood	Increased IL-6, IL-8, TNF-α, IFN-γ, and CRP levels	Low-grade systemic inflammation Modulation of nociceptor sensitivity and synaptic plasticity Neuroendocrine dysregulation contributing to widespread pain, fatigue, and sleep disturbances	[45,46,47,48]
	Increased S100A8, S100A9, VCAM1, CD163, SERPINA1, and ANXA1 transcripts	Chronic immune activation Dysregulated leukocyte function Potential impact on neuroimmune communication across the BBB	[49,50]
	Elevated AXIN1, STAMBP, and SIRT2, together with increased chemokine levels (CCL2, CCL17, CXCL9, and CXCL11)	Immune modulation, associated with symptom severity and disease burden	[22,51]

**Table 2:** Key findings and implicated mechanisms identified by omics approaches in metabolites, transcriptomics, and pathways related to metabolism, inflammation, and neurotransmission. Abbreviations: BCAAs (branched-chain amino acids), GABA (gamma-aminobutyric acid), TCA (tricarboxylic acid), TLR (Toll-like receptor), and ATP (adenosine triphosphate).

Omic Technology	Major Targets	Main Findings	Implicated Mechanisms	References
Metabolomics	Aminoacids	Alterations in amino acid profiles: BCAAs, aromatic amino acids, glutamine/glutamate ratio, and GABA and arginine levels	Disruptions in energy metabolism, neurotransmission, neuroinflammation, and central sensitization	[79,80,81,82,83,84,85,86,87,88,89,90,91,92,93,94,95,96,97]
	Lipids	Elevated ceramides, sphingomyelins, phosphatidylcholines, acylcarnitines, and eicosanoids Increased oxidative stress markers	Mitochondrial dysfunction, apoptosis, neuroinflammation, and impaired lipid metabolism	[88,89,90,91,92]
	Energy and purine metabolism	Dysregulated TCA cycle, elevated lactate/pyruvate ratio, altered purines, and creatine/creatinine imbalance	Mitochondrial dysfunction, oxidative stress, and energy deficiency	[93,94,95,96]
Transcriptomics	Immune system	Increased pro-inflammatory cytokines Reduced anti-inflammatory mediators Upregulated TLR gene expression	Chronic inflammation	[97,98,99]
	Energy metabolism	Dysregulated mitochondrial, glycolytic, and fatty acid oxidation genes	Impaired ATP production	[100,101,102]
	Oxidative stress response	Dysregulated oxidative stress-response genes	Activation of oxidative stress-response pathways	[103]
	Neurotransmission	Downregulated inhibitory neurotransmission genes Altered circadian/stress-response genes	Excitatory-inhibitory imbalance, hyperalgesia, and sleep disturbances	[104,105]

## Data Availability

Not applicable. No new data were generated.

## Abbreviations

The following abbreviations are used in this manuscript:

ACC	Anterior cingulate cortex
ANXA1	Annexin A1
ASICs	Acid-sensing ion channel
AXIN1	Axis inhibition protein 1
BBB	Blood–brain barrier
BCAA	Branched-chain amino acid
BDNF	Brain-derived neurotrophic factor
C16:0	Ceramide with a 16-carbon fatty acid and 0 double bonds (palmitic acid)
C18:0	Ceramide with an 18-carbon fatty acid and 0 double bonds (stearic acid)
C20:0	Ceramide with a 20-carbon fatty acid and 0 double bonds (arachidic acid)
CAR	Cortisol awakening response
CBT	Cognitive-behavioral therapy
CCL11	C-C motif chemokine ligand 11
CCL17	C-C motif chemokine ligand 17
CCL2	C-C motif chemokine ligand 2
CCL5	C-C motif chemokine ligand 5
CD163	Cluster of differentiation 163
CNS	Central nervous system
COMT	Catechol-O-methyltransferase
COX	Cyclooxygenase
CPT1	Carnitine palmitoyltransferase 1
CRH	Corticotropin-releasing hormone
CRP	C-reactive protein
CSF	Cerebrospinal fluid
CX3CL1	C-X3-C motif chemokine ligand 1
CXCL10	C-X-C motif chemokine ligand 10
CXCL5	C-X-C motif chemokine ligand 5
CXCL8	C-X-C motif chemokine ligand 8
CXCL9	C-X-C motif chemokine ligand 9
CyB	Cytochrome b
DMN	Default mode network
DTI	Diffusion tensor imaging
EMA	Ecological momentary assessment
fMRI	Functional magnetic resonance imaging
GABA	Gamma-aminobutyric acid
GABRB3	Gamma-aminobutyric acid type A receptor beta3 subunit
GEP	Graded exercise program
GPX	Glutathione peroxidase
GR	Glucocorticoid receptor
HO-1/HMOX1	Heme oxygenase 1
HPA	Hypothalamic–pituitary–adrenal axis
HRV	Heart rate variability
IBS	Irritable bowel syndrome
IFN-γ	Interferon gamma
IL-10	Interleukin 10
IL-17	Interleukin 17
IL-1β	Interleukin 1 beta
IL-6	Interleukin 6
IL-8	Interleukin 8
LDH	Lactate dehydrogenase
LOX	Lipoxygenase
LPC	Lysophosphatidylcholine
LPC 16:0	Lysophosphatidylcholine with a 16-carbon fatty acid (palmitic acid)
LPC 18:0	Lysophosphatidylcholine with an 18-carbon fatty acid (stearic acid)
MBSR	Mindfulness-based stress reduction
MDA	Malondialdehyde
ML	Machine learning
MRS	Magnetic resonance spectroscopy
mTOR	Mammalian target of rapamycin
MyD88	Myeloid differentiation primary response 88
ND4	NADH dehydrogenase subunit 4
NOS	Nitric oxide synthase
NQO1/NAD(P)H	Quinone dehydrogenase 1
NR3C1	Nuclear receptor subfamily 3 group C member 1
NREM	Non-rapid eye movement
NRF-1	Nuclear respiratory factor 1
OSI	Oxidative stress index
P2X	Purinergic P2X receptors
P2Y	Purinergic P2Y receptors
PAG	Periaqueductal gray
PBMC	Peripheral blood mononuclear cell
PC	Phosphatidylcholine
PC 34:1	Phosphatidylcholine with a total of 34 carbons in its two fatty acyl chains and 1 double bond
PC 36:2	Phosphatidylcholine with 36 carbons in total and 2 double bonds
PET	Positron emission tomography
PFC	Prefrontal cortex
PGE2	Prostaglandin E2
QST	Quantitative sensory testing
REM	Rapid eye movement
ROS	Reactive oxygen species
S100A8	S100 calcium-binding protein A8
S100A9	S100 calcium-binding protein A9
SERPINA1	Serpin family A member 1
SIRT2	Sirtuin 2
SM	Sphingomyelin
SM d18:1/16:0	Sphingomyelin with a sphingosine backbone of 18 carbons and 1 double bond + 16-carbon fatty acid (palmitic acid)
SM d18:1/18:0	Sphingomyelin with a sphingosine backbone of 18:1 + 18-carbon fatty acid (stearic acid)
SNRI	Serotonin-norepinephrine reuptake inhibitor
SOCS3	Suppressor of cytokine signaling 3
SOD	Superoxide dismutase
STAMBP	STAM binding protein
TAS	Total antioxidant status
TCA	Tricarboxylic acid
Tfam	Mitochondrial transcription factor A
TGF-β	Transforming growth factor beta
TLR2	Toll-like receptor 2
TLR4	Toll-like receptor 4
TNF-α	Tumor necrosis factor alpha
TOS	Total oxidant status
TRPV	Transient receptor potential vanilloid
UCP2	Uncoupling protein 2
VBM	Voxel-based morphometry
VCAM1	Vascular cell adhesion molecule 1
